# Redox-active tetrathiafulvalene and dithiolene compounds derived from allylic 1,4-diol rearrangement products of disubstituted 1,3-dithiole derivatives

**DOI:** 10.3762/bjoc.6.113

**Published:** 2010-10-21

**Authors:** Filipe Vilela, Peter J Skabara, Christopher R Mason, Thomas D J Westgate, Asun Luquin, Simon J Coles, Michael B Hursthouse

**Affiliations:** 1 WestCHEM, Department of Pure and Applied Chemistry, University of Strathclyde, 295 Cathedral Street, Glasgow, G1 1XL, UK; 2 School of Chemistry, University of Manchester, Oxford Road, Manchester, M13 9PL, UK; 3 Departamento de Química Inorgánica, Instituto de Ciencia de Materiales de Aragón, Universidad de Zaragoza-C.S.I.C., 50009, Zaragoza, Spain; 4 Department of Chemistry, University of Southampton, Highfield, Southampton, SO17 1BJ, UK

**Keywords:** aldehydes, metal-coordination, rearrangement, sulfur heterocycles, tetrathiafulvalene

## Abstract

We present a series of compounds by exploiting the unusual 1,4-aryl shift observed for electron-rich 1,3-dithiole-2-thione and tetrathiafulvalene (TTF) derivatives in the presence of perchloric acid. The mechanistic features of this rearrangement are discussed since this synthetic strategy provides an alternative route for the synthesis and functionalisation of sulfur rich compounds including redox active compounds of TTFs, and a Ni dithiolene.

## Introduction

**Figure 1:**
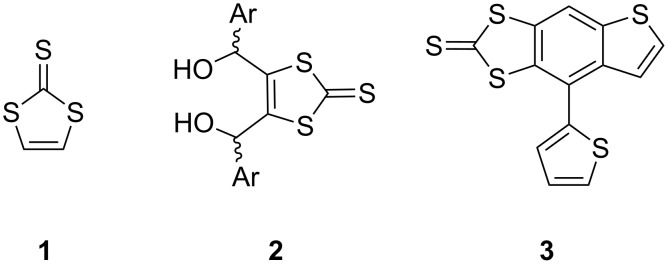
Chemical structures of compounds **1**–**3**.

The search for novel structures derived from TTF has led to extensive investigations of the chemistry of 1,3-dithiole-2-thione (**1**, Figure 1) [1–2], since this heterocycle and its derivatives are frequently used as convenient precursors to TTF compounds through phosphite-mediated coupling [3].

Our efforts to prepare triaryl derivatives of compound **1** led to the discovery of an unexpected rearrangement of 4,5-bis(2-arylhydroxymethyl)-1,3-dithiole-2-thiones (**2**) in the presence of acid catalysts [4]. As well as the expected dihydrofuran, formed by a nucleophilic ring-closing reaction, other compounds were also produced depending on the nature of the aryl group, the nature of the solvent and the acid used (e.g., **3**). The extent to which the aryl groups and their substituents are able to stabilise the carbocation, formed by the loss of a protonated hydroxy group, is key in determining the reaction product. The diverse range of π-rich structures that can be obtained from the diol includes ketone, aldehyde and haloalkene functionalities and these offer further possibilities for the synthetic use of TTF-based compounds that are well known in conducting materials [5].

Herein, we report an extended study of these unusual 1,4-aryl rearrangements involving 1,3-dithiole-2-thione and TTF derivatives, postulate a likely reaction mechanism and comment on substituent effects.

## Results and Discussion

### Synthesis

**Scheme 1:**
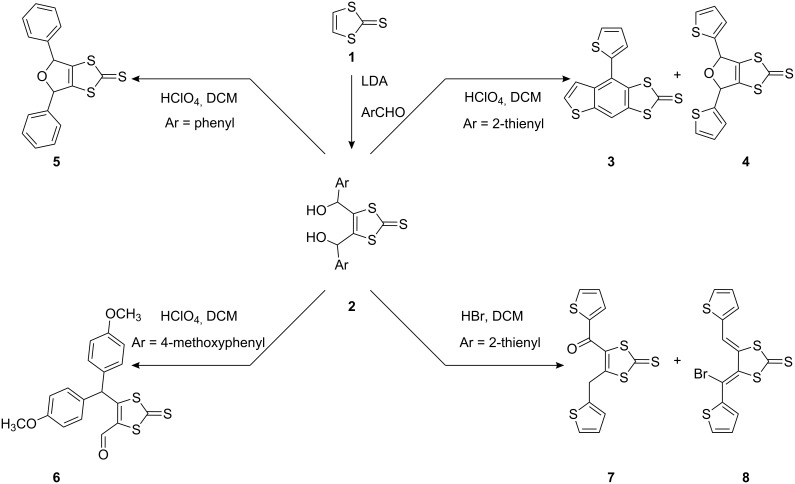
Acid-catalysed behaviour of 4,5-bis(2-arylhydroxymethyl)-1,3-dithiole-2-thiones **2**.

Scheme 1 summarises the overall procedure that leads to the 1,4-aryl rearrangements. The lithiation of compound **1** with LDA and subsequent reaction with aryl carboxaldehydes affords the corresponding bisalcohol products **2** in good yield (75–90%). Under ambient conditions, these diols decompose slowly. However, on treatment with a few drops of perchloric acid to solutions of **2** in dichloromethane (CH_2_Cl_2_), the rate of decomposition of the starting materials greatly increases and in all cases the diol is consumed within 24 h. Dihydrofurans, such as **5** (87% yield), were isolated from the corresponding diols as mixtures of stereoisomers, whereas the 4-methoxyphenyl substituted diol gave the rearrangement product **6** in 44% yield. The rearrangement products from **2** were all obtained as the major product (by tlc). By contrast, when **2** was treated with HBr in CH_2_Cl_2_, compound **7** was isolated as a yellow oil via the initially produced dihydrofuran **4** along with compound **8** (observed only by ^1^H NMR) [6].

The behaviour of 4,5-bis(2-thienylhydroxymethyl)-1,3-dithiole-2-thione in the presence of perchloric acid is of particular interest. Under these conditions the diol undergoes an intramolecular rearrangement to form the ring-fused product **3** in good yield [6].

### Discussion

**Scheme 2:**
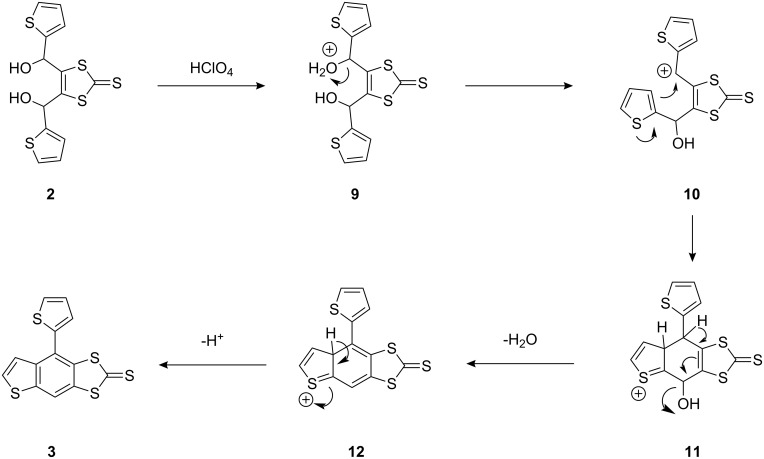
The proposed mechanism for the formation of **3**.

The first step in this process is the protonation of one of the hydroxy groups (Scheme 2). Subsequent loss of water affords a carbocation that can be stabilised by resonance of π-electrons from either the 1,3-dithiole-2-thione or thienyl groups. This intermediate can then undergo intramolecular nucleophilic ring-closure by the remaining hydroxy group with the formation of the dihydrofuan **4** shown in Scheme 1. Dominating this process is the competing electrophilic attack of the allylic carbocation at the 3-position of the thiophene ring bearing the carbinol group, forming a new C–C bond and producing a 6-membered ring. Concomitantly, the cationic centre migrates to produce a C=S^+^ form, where it is stabilised by π-electron density. The second hydroxy group is eliminated in a dehydration process. Loss of a further proton affords the neutral product **3** with a fully aromatised 6-membered ring.

This process bears similarities to other rearrangements that are well known in classical organic chemistry [7–9]. The pinacol rearrangement, for example, also involves the loss of H_2_O from a protonated diol followed by migration of the cation to yield a protonated carbonyl group and simultaneous rearrangement of the C–C skeleton. The principle of intermediate carbocation stabilisation also involves the breaking of carbon–carbon or carbon–hydrogen bonds followed by formation of new ones via alkyl, hydride and allylic shifts.

However, one cannot assume that the mechanism is pinacol-like. Saturated 1,4-diols favour the formation of tetrahydrofuran derivatives, whilst allylic cations will involve some degree of stabilisation through hyperconjugation and delocalisation of the positive charge, which could complicate the pinacol mechanism. Related reactions of allylic alcohols are not unknown: periodic acid has been shown to oxidise one of the alcohol groups in a 1,4-diol fragment to yield the corresponding aldehyde, without any further change to the remnant allylic alcohol [10]; a related mechanism has been reported for a self-coupling intermolecular reaction, which involves an intermediate allylic cation generated under acidic conditions from an electron-rich thienylmethanol derivative, where no intramolecular rearrangement took place [11]. Also worth noting, is a recent study on cationic 1,4-aryl migration observed during reactions of vinylidenecyclopropanes with electron-rich bis(*p*-alkoxyphenyl)methanols [12].

**Scheme 3:**
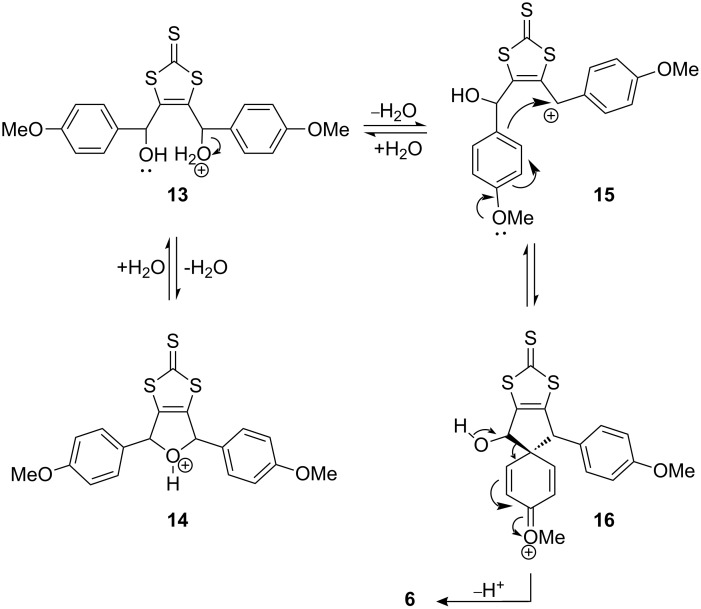
The proposed mechanism for the decomposition of **13** in the presence of perchloric acid.

We have extended our study using a 4-methoxyphenyl substituted diol as an example. The proposed decomposition of **13** is described in Scheme 3. It should be noted that the involvement of a phenonium intermediate in aryl shifts has been previously observed [13], and that theoretical studies have also shown that there is significant stabilisation of the phenonium ion through back-bonding from the ethylene unit of spirocyclopropyl benzenium species [14].

However, as seen in Scheme 3, we propose a methoxy stabilised spirocyclopentyl benzenium intermediate. This mechanism is only valid for the 4-methoxy derivative as the position of the electron donating groups is fundamental in producing the dihydrofuran or the 1,4-shift products.

It is also unusual that the sole product from the corresponding 2-methoxyphenyl compound leads to a dihydrofuran derivative, particularly since the electron donating substituent effect should be identical to that of the 4-methoxy compound, which rearranges to give the corresponding dihydrofuran. One possible explanation for this can be derived from the X-ray crystal structure of the dihydrofuran derivative [4]. The intramolecular close contacts between O(2)···S(2) and O(3)···S(3) arise from a three-centre four-electron interaction [15], which decreases the electron releasing ability of the methoxy groups towards the benzene ring, thereby conferring a weaker stabilising effect upon the phenonium ion. This type of intramolecular interaction has been observed in the X-ray crystal structures of other triaryl dithiole-2-thione species [16].

**Figure 2:**
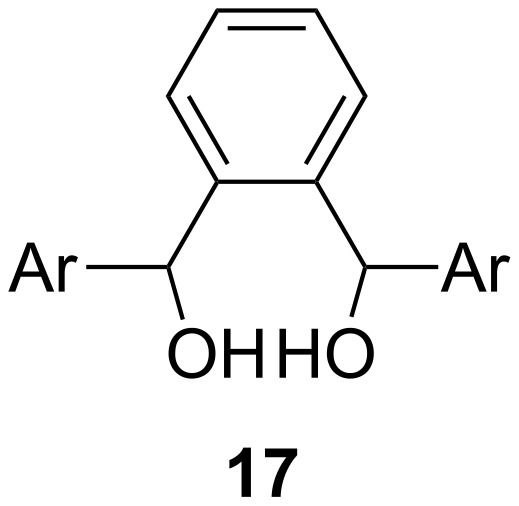
Generalised structure of diol **17**.

In order to investigate the limitations of this new rearrangement process, we carried out studies on analogous benzene derivatives (**17**, Figure 2). In each case, the addition of perchloric acid resulted in a complex mixture of products, which could not be easily separated [4].

**Scheme 4:**
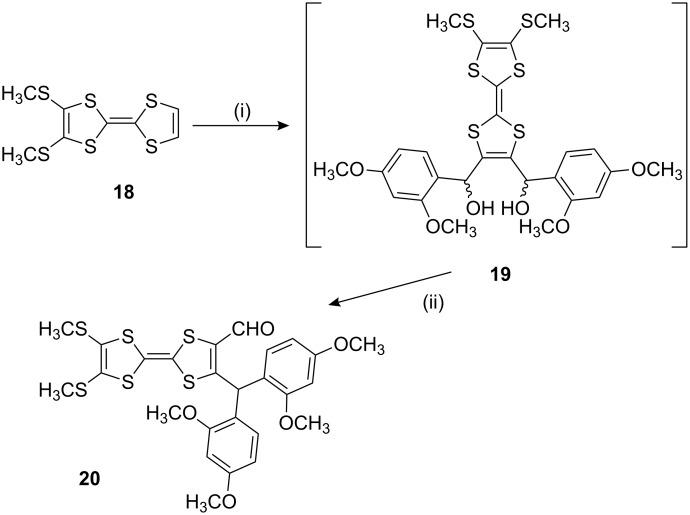
Reagents and conditions: (i) LDA (1 equiv), 2,4-dimethoxybenzaldehyde (1 equiv), then repeat, −78 °C, THF; (ii) HClO_4_, CH_2_Cl_2_, rt.

The 1,4-aryl shift was also attempted on compound **19** to ascertain whether or not this type of rearrangement is accessible to TTF derivatives (Scheme 4). The diol **19** was prepared directly from compound **18** [17] and treated with two equivalents of LDA and 2,4-dimethoxybenzaldehyde, as described above for compound **1**. The addition of perchloric acid to a crude sample of the diol resulted in a mixture of products, however, the TTF derivative **20** was isolated by column chromatography in only 9% overall yield from **18**. Since the reaction of tetrathiafulvalenyllithium with simple aldehydes proceeds to give the corresponding alcohols in 40–60% yield [18], the rearrangement of **19** to give **20** is estimated to proceed in 25–55% yield. Cyclic voltammetry (in dichloromethane, vs Ag/AgCl) showed two sequential, reversible oxidation steps for compound **20** at *E*^1/2^ = +0.72 and +1.09 V.

**Scheme 5:**
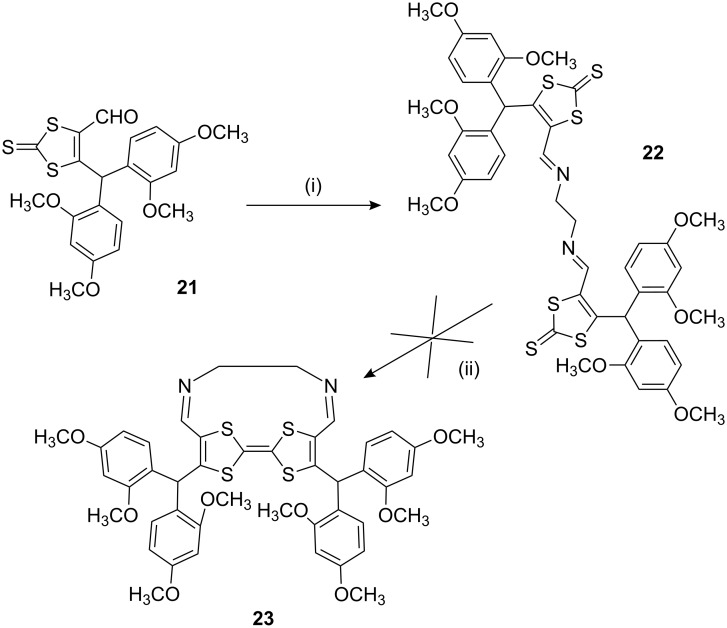
Reagents and conditions: (i) ethylenediamine, AcOH, MeOH; (ii) P(OEt)_3_, 120 °C, 3 h.

To demonstrate the reactivity of the 1,4-shift products and to assess the degree of steric hindrance by the aromatic units towards the formyl group, compound **21** [4] was reacted with ethylenediamine in the presence of a catalytic amount of acetic acid (Scheme 5).

**Figure 3:**
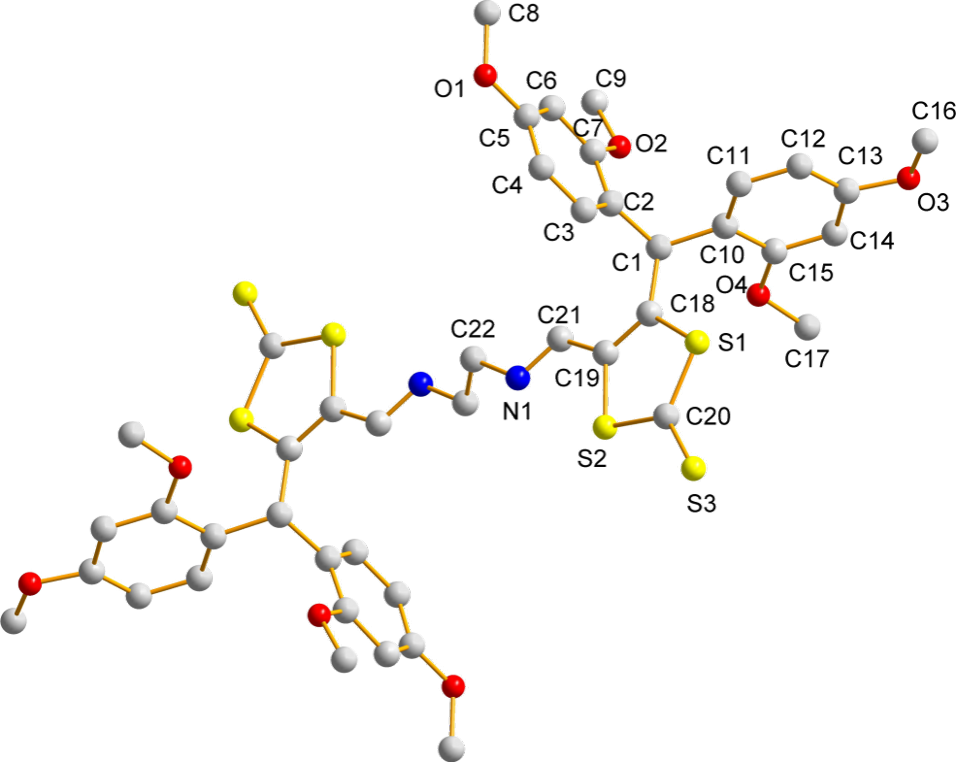
Molecular structure and numbering scheme of compound **22** with Hs omitted.

The Schiff base product **22** was obtained in 62% yield as a pale brown crystalline solid. Although highly strained and buckled TTF derivatives have been reported [19], treatment of **22** with triethyl phosphite did not give the desired self-coupled TTF compound **23**. The X-ray structure of **22** is shown in Figure 3. The molecule possesses an inversion centre located at the C(22)–C(22’) bond. A close intramolecular contact is observed between S(2)···N(1) at 2.936(3) Å. This interaction serves to strengthen the degree of conjugation between the imine functionality and the dithiole unit: the torsion angle within C(18)–C(19)–C(21)–N(1) is 173.2(9)° whilst the bond length of C(19)–C(21) (1.449(6) Å) is shorter than that of a typical conjugated single C(sp^2^)–C(sp^2^) bond (mean 1.455 Å) and substantially shorter than non-conjugated ones (1.478 Å) [20].

### Redox-active derivatives of compound 3

**Scheme 6:**
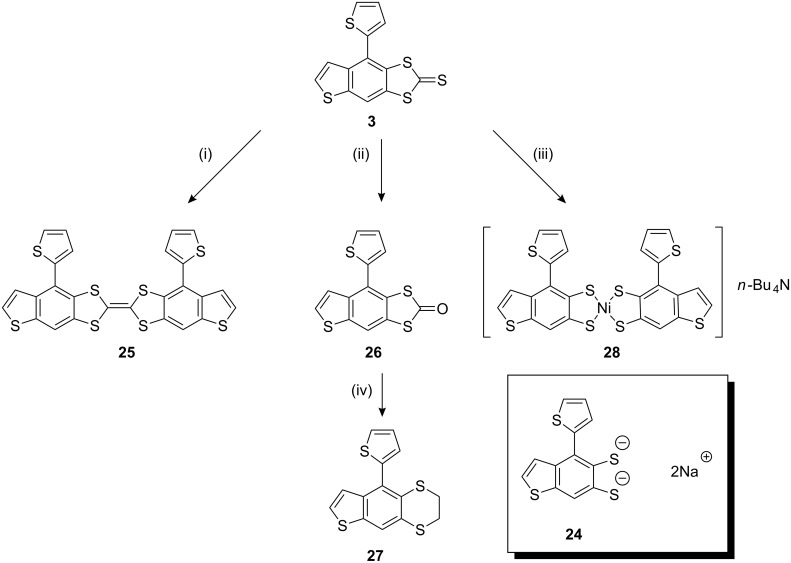
Reagents and conditions: (i) P(OEt)_3_, reflux; (ii) Hg(OAc)_2_, CH_2_Cl_2_/AcOH; (iii) NaOEt, THF, reflux, then Bu_4_NBr and NiCl_2_·6H_2_O, rt; (iv) NaOMe, THF, then 1,2-dibromoethane.

Compound **3** contains a fused thienobenzodithiole-2-thione unit. These three annelated cyclic moieties present an interesting and potentially useful structural motif for materials applications. For example, benzyl and thienyl annelated TTF derivatives and analogues have been shown to perform well as semiconductor materials in organic field effect transistors (OFETs) [21–22]. This has been attributed to the enhanced π–π stacking of the extended aromatic groups, which improves intermolecular electronic transfer. Annelated aromatic groups also lower the electron donating ability of TTF, which improves resistance to oxidative degradation in field effect transistor (FET) devices, whilst the incorporation of solubilising groups to these systems offers good processability for the annelated products [23–25]. The thione group in **3** has the potential for several synthetically useful manipulations (Scheme 6).

Compound **3** was shown to be a synthetically versatile compound. Refluxing of **3** in triethyl phosphite followed by purification by filtration through silica gel (eluting with chloroform) gave a mixture of geometrical (*cis-* and *trans*-) isomers of the TTF derivative **25** in 12% yield. The two isomers could not be separated either by chromatography or by recrystallisation. It should be noted, that in the oxidation of **25** the central C=C bond is broken, allowing free rotation and therefore isomerisation. It can be anticipated that the two isomers will not differ significantly in their electrochemical properties (see below).

Compound **3** was trans-chalcogenated by mercury(II)acetate in a mixture of acetic acid and CH_2_Cl_2_ to give the oxo derivative **26** in 89% yield. Purification of **26** was achieved by recrystallisation from CH_2_Cl_2_ and petroleum ether. Treatment of **26** with methoxide solution (generated in situ) resulted in sequential nucleophilic addition-elimination at the carbonyl carbon, generating the disodium dithiolate salt **24**.

**Figure 4:**
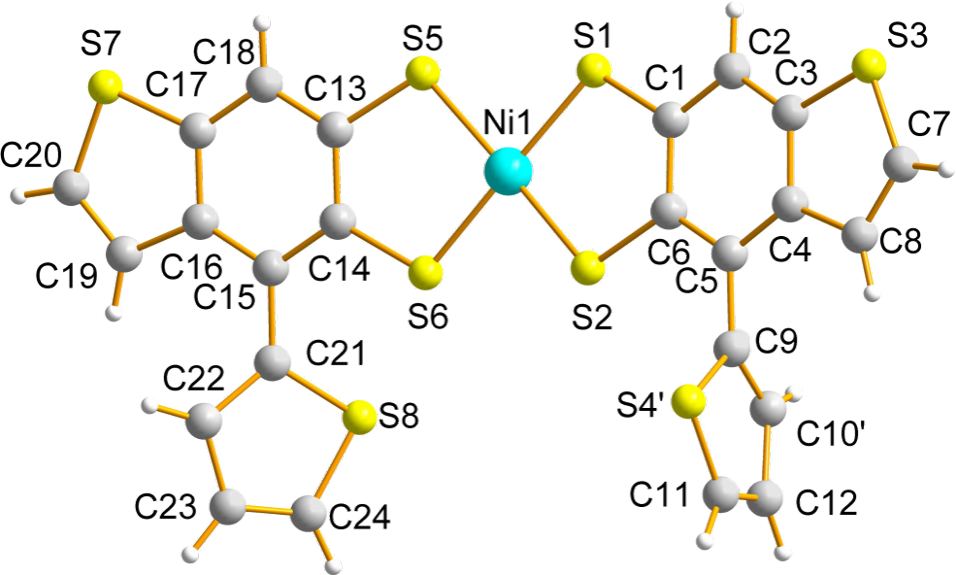
Molecular structure of **28** with the tetrabutylammonium cation omitted.

**Figure 5:**
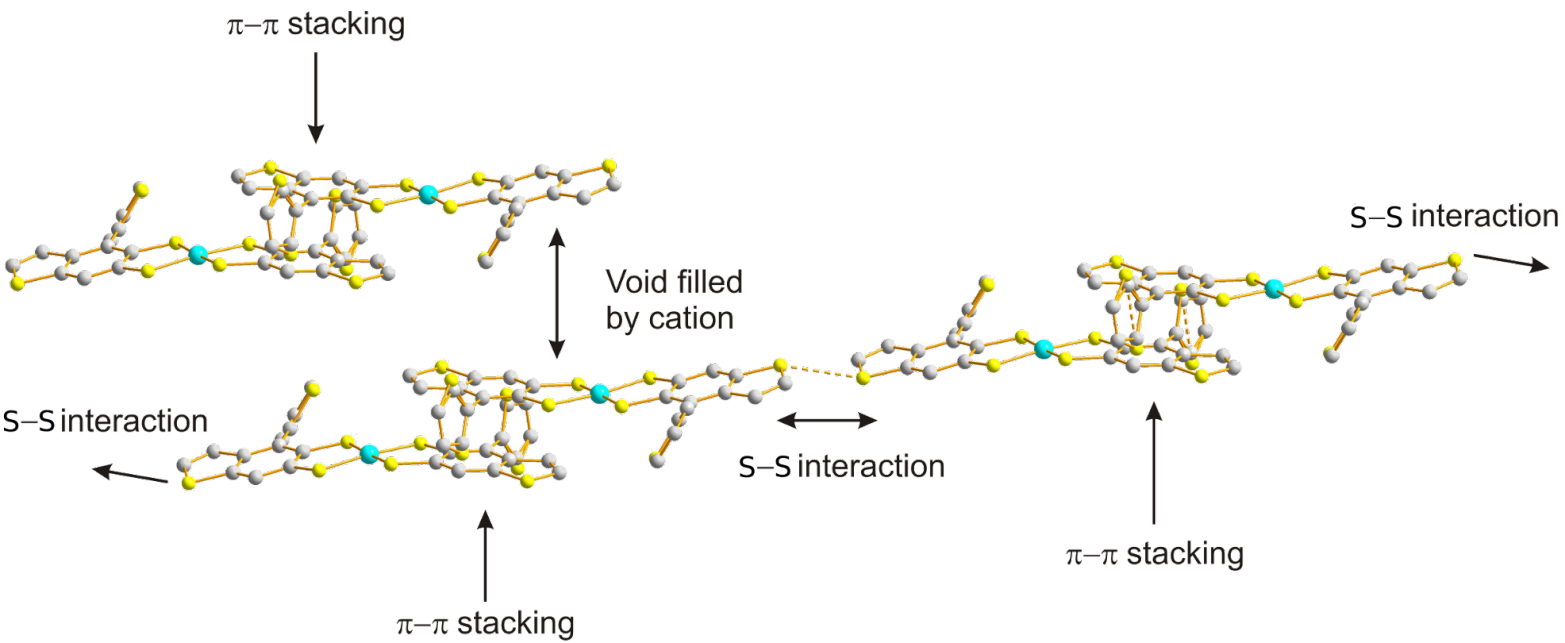
Packing diagram of **28** identifying close intermolecular contacts.

The dianion was subsequently ring-closed by two S_N_2 reactions with 1,2-dibromoethane in THF to yield **27**(56% yield) after purification by column chromatography (eluting with 1:1 (v/v) CH_2_Cl_2_:petroleum ether). Dithiolate **24** was also generated from the analogous reaction with **3**. On this occasion, treatment of **24** with excess nickel(II)chloride and tetrabutylammonium bromide afforded the nickel(II)dithiolato complex **28** (40% yield) after purification by recrystallisation from CH_2_Cl_2_ and hexane. Dithiolenes represent an important and well-studied class of molecular conductors [26] and we have previously studied thiophene-containing analogues as precursors to highly electroactive polythiophenes [27–28]. We were interested in the self-assembly of compound **28** in the solid state to assess whether or not this material would be suitable as a semiconductor in OFETs [29]. Single crystals of compound **28** were grown from a dichloromethane/hexane solution. X-ray crystallographic data confirmed that the isolated nickel dithiolene was monoanionic and that only one of the two possible isomers of this product was isolated by the recrystallisation method (both non-fused thiophenes are on the same side of the fused framework). The molecular structure of the compound is shown in Figure 4 and the packing diagram is shown in Figure 5. The peripheral, non-fused thiophenes are significantly distorted from the plane of the fused skeleton, leaving them weakly conjugated to the main π-framework. The torsion angles for C22–C21–C15–C14 and C10–C9–C5–C6 are 133.97(24)° and 81.79(60)°, respectively. There is a slight distortion in the Ni-dithiolene core, which is commonly planar, with an angle of 6.45(2)° between the two planes of the component rings. There is some disorder in one of the peripheral thiophene rings, with atoms S4 and C10 occupying common positions with 50% probability.

The packing diagram in Figure 5 highlights the intermolecular interactions that exist in the bulk, which in turn would support semiconductor behaviour. Firstly, there are sulfur–sulfur close contacts between adjacent S7 atoms (3.2479(8) Å), and these interactions are restricted to dimers. Secondly, the six-membered ring comprising–C1–C2–C3–C4–C5–C6– and the adjacent thiophene (–S3–C7–C8–C4–C3–) form π–π interactions with a neighbouring molecule, such that the overlap between the corresponding molecules is restricted to one minor portion of the molecule. The centroid–centroid distance between the benzene and thiophene rings is 3.74 Å and this interaction is also constrained to dimers. Although in Figure 5 the cations have been omitted for clarity, the diagram shows where the tetrabutylammonium molecules reside to block further close π–π interactions. These interactions ultimately lead to orbital overlap in one dimension, albeit through a combination of chalcogen–chalcogen and π–π contacts.

**Figure 6:**
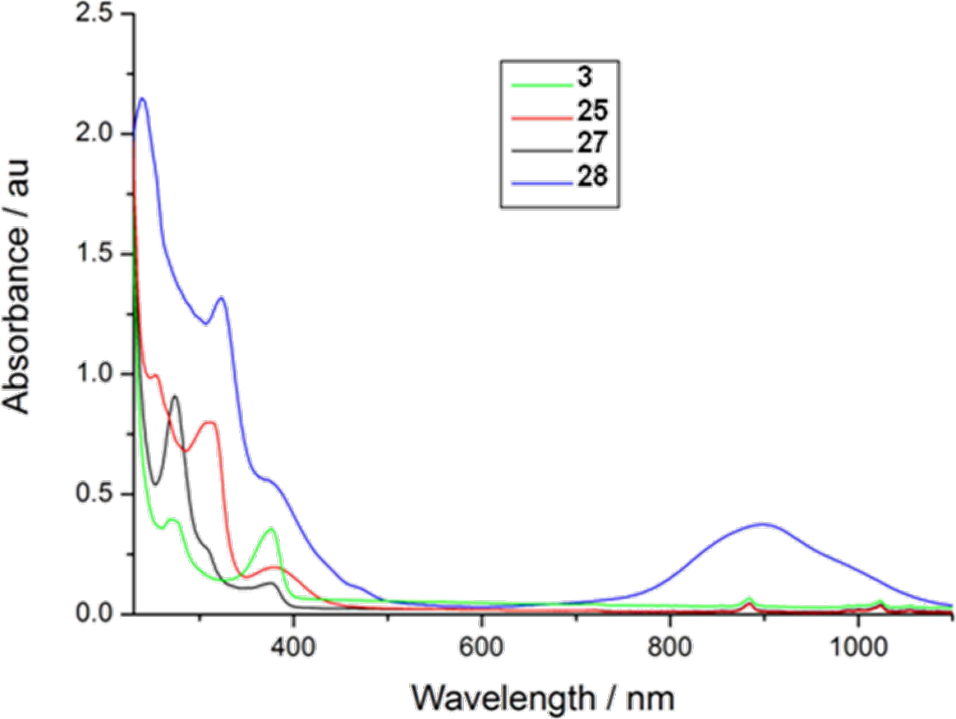
UV–visible spectra of **3**, **25, 27** and **28** in CH_2_Cl_2_ solution.

The UV–visible electronic absorption spectra of compounds **3**, **25, 27** and **28** were recorded in CH_2_Cl_2_ solution and are shown in Figure 6. The spectra of thione **3** and ethylenedithio-bridged derivative **27** show similar features. Each shows a peak at 376 nm and one around 275 nm. The intensities of these peaks are reversed in each case, however. In **27** the 376 nm peak is far less prominent compared to the 275 nm peak than in the thione. The spectrum of **27** also shows a shoulder at around 310 nm. These two bands are also present in TTF derivative **25** and the nickel complex **28**.

The band at 376 nm in **3** and **27** appears at 382 nm in **25** and is seen as a shoulder at approximately 372 nm in **28**. The higher energy band at around 275 nm in **3** and **27** is shifted in **25** and **28** to lower energy (313 nm in **25**, 324 nm in **28**). The spectrum of **28** also shows a broad feature at around 900 nm, corresponding to the π–π * transition of the highly delocalised nickel dithiolene unit.

**Figure 7:**
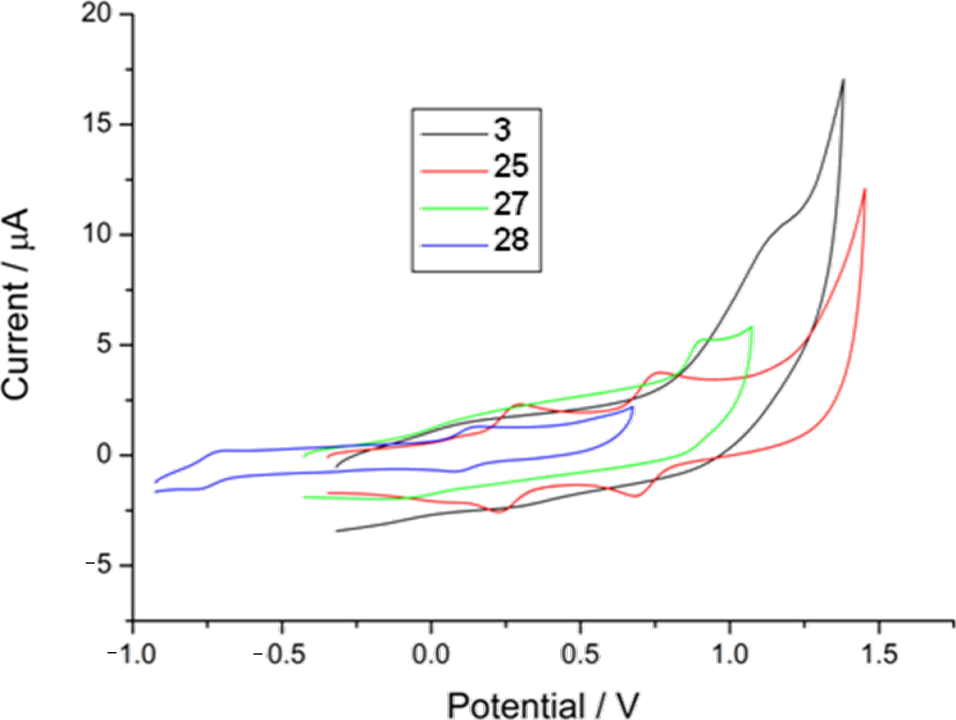
Cyclic voltammograms of compounds **3**, **25**, **27**, and **28**. Glassy carbon working electrode, using Pt wire counter and Ag/AgCl reference electrodes, in CH_2_Cl_2_ (substrate ca. 10^−3^ M), Bu_4_NPF_6_ supporting electrolyte (0.1 M), with a scan rate of 100 mV s^−1^.

The electrochemistry of **3**, **25**, **27** and **28** was investigated by cyclic voltammetry. Solutions of the compounds (0.1 mM) were prepared in anhydrous CH_2_Cl_2_ with 0.1 M tetrabutylammonium hexafluorophosphate as the supporting electrolyte. Glassy carbon, platinum wire, and Ag/AgCl electrodes were used as the working, counter and reference electrodes, respectively. The cyclic voltammograms are shown in Figure 7, and the potential values of the redox processes are collated in Table 1.

**Table 1:** Redox potentials of compounds **3**, **25**, **27** and **28** (half-wave potentials except where irreversible).

	**3**	**25**	**27**	**28**

**E^½ ^_1_ (mV)**	+1170 (irreversible)	+260	+900 (irreversible)	−740
**E^½ ^_2_ (mV)**	—	+720	—	+110

In compounds **3** and **27** an irreversible single electron oxidation wave is seen at +1170 mV and +900 mV, respectively. Further oxidation processes of these materials do not occur within the electrochemical window of the solvent. The electron withdrawing effect of the thione causes the oxidation to occur at a higher potential in **3** than in **27**. In TTF derivative **25** the two characteristic reversible single electron redox waves, arising from the step-wise formation of the TTF radical cation and dication, are seen at +260 mV and +720 mV, respectively. The formation of the TTF dication hinders subsequent removal of an electron from the thiophene units, so further oxidations are beyond the electrochemical window of the solvent. Compound **28** is reversibly oxidised to **28**^+·^ at −740 mV, within the range seen in other nickel dithiolene complexes. A second single-electron oxidation is seen at around +110 mV, and appears to be reversible.

Attempts to electro-polymerise compounds **3**, **25**, **27** and **28** were unsuccessful. The primary reason for this may be the inability to oxidise the fused thiophene ring, ruling out coupling through the 2-position of this unit. Electro-oxidative coupling of **3** and **27** is therefore only accessible through the pendant group making a dimer the only possible product. Compounds **25** and **28** have two oxidisable pendant thiophene groups, so polymerisation could theoretically proceed through the coupling of these units. However, repeated cycling over the oxidative electroactive range of **25** and **28** did not produce the sequential increase of current characteristic of the formation of a polymer film on the working electrode. Furthermore, no deposited material was observed on the working electrode surface. Coupling of **25** and **28** through the pendant thiophenes may be sterically demanding, but the formation of short oligomers cannot be ruled out in this mechanism.

## Conclusion

We have shown that the unusual 1,4-aryl shift observed in the presence of perchloric acid for electron-rich 1,3-dithiole-2-thione and TTF derivatives can be exploited to further expand this family of compounds. For example, previous work has shown that the electron-donating ability of terthiophenes can be tailored by this approach [30–31]. Also, the metal-coordination properties of the dithiolate anion, as shown in compound **28**, present a potentially rich avenue for further investigation by variation of the metal cation. This dianion could also be reacted with a variety of dihaloalkanes to create a family of compounds with alkyl bridges of different lengths. Finally, this reaction is important for understanding the stabilising effects of substituents upon the allylic cation and, independently, the phenonium intermediate, as well as providing an alternative strategy towards the functionalisation of benzylated 1,3-dithiol-2-thione and TTF derivatives.

## Experimental

### X-ray crystallography

Table 2 provides all the data collected and the refinement parameters for the structures of **22** and **28**. All data were collected at 150 K, on a Nonius KappaCCD area detector diffractometer, at the window of a Nonius FR591 rotating anode (λ MoK_α_ = 0.7106 Å). A correction was applied to account for absorption effects by means of comparing equivalent reflections, using the program SORTAV [32–33]. A solution was obtained via direct methods and refined [34] by full*-*matrix least-squares on F^2^, with hydrogens included in idealised positions. There is rotational disorder of a thiophene moiety in **28**, where C10 and S4 occupy the same position in the ring with a site occupancy of 50%. Supplementary data for **22** and **28** have been deposited with the CCDC; deposition numbers = CCDC 216240 and 778197, respectively. These data can be obtained free of charge at www.ccdc.cam.ac.uk/conts/retrieving.html (or from the Cambridge Crystallographic Data Centre, 12, Union Road, Cambridge CB2 1EZ, UK; fax: (internat.) +44-1223/336-033; E-mail: deposit@ccdc.cam.ac.uk).

**Table 2:** Data collection and refinement parameters for structures **22** and **28**.

	**22**	**28**

Empirical formula	C_44_H_44_N_2_O_8_S_6_	C_40_H_48_NNiS_8_
Molecular mass	921.17	857.98
Crystal system	*P*2_1_/*c*	*P*-1
Space group	Monoclinic	Triclinic
*a*/Å	12.185(2)	9.9368(2)
*b*/Å	10.904(2)	10.0005(2)
*c*/Å	17.597(3)	22.5310(3)
α/°	90	95.050(2)
β/°	106.87(3)	93.054(2)
γ/°	90	114.388(2)
V/Å^3^	2237.3(8)	2021.34(4)
*Z*	2	2
*D*_calcd._/g cm^−3^	1.367	1.410
*µ*/mm^–1^	0.360	0.924
Crystal size/mm^3^	0.25 × 0.25 × 0.20	0.38 × 0.04 × 0.01
*θ* range	3.04–25.01	3.01–27.50
Measured reflections	15332	55561
Independent reflections [*I* > 2*σ*(*I*)]	3918	8113
*R*_int_	0.0672	0.0408
*R* indices [*F*^2^ > 2*σF*^2^]	0.0664/0.1768	0.0383/0.0976
*R* indices [all data]	0.0865/0.1954	0.0454/0.1018
Goodness of fit	1.009	1.039
*ρ*_max_/*ρ*_min_/e Å^−3^	0.877/−0.497	0.714/−0.555

### General

Unless, otherwise stated all reactions were performed under an inert atmosphere of dry nitrogen using standard Schlenk techniques. All glassware was flame dried under vacuum prior to use. All solvents and reagents were purchased from the Sigma-Aldrich chemical company and used as received. ^1^H, and ^13^C NMR spectra were recorded on a Bruker AC300 FTNMR instrument operating at room temperature (300 MHz for ^1^H, 75 MHz for ^13^C). Mass spectra were recorded on a Kratos concept 1S instrument. Infrared spectra were recorded on a Specac single reflectance ATR instrument (4000–400 cm^−1^, resolution 4 cm^−1^). Elemental analysis was performed by the University of Manchester micro-analytical laboratory. Melting points were recorded on a Gallenkamp melting point apparatus and are uncorrected. Cyclic voltammetry was performed with a CH instruments voltammetric analyser with *iR* compensation, in anhydrous dichloromethane, with aqueous Ag/AgCl as the reference electrode and platinum wire and gold disk as the counter and working electrodes, respectively. The solution was degassed (N_2_) and contained the substrate in a concentration of ca. 10^−3^ M together with Bu_4_NPF_6_ (0.1 M) as the supporting electrolyte.

#### General procedure for compounds **5**–**6** and **20**

To a solution of vinylene trithiocarbonate or compound **15** in dry tetrahydrofuran (20–80 ml) at −78 °C under nitrogen, was added lithium diisopropylamide mono(tetrahydrofuran) (1.5 M in hexanes, 1.1 equiv). The mixture was stirred under dry nitrogen for 20 min. The relevant aldehyde was then added and, after stirring for 5 min, a second portion of LDA was added (1.1 equiv). The mixture was stirred for a further 15 min followed by the addition of another portion of aldehyde and stirred for a another 5 min. The reaction was then allowed to warm to room temperature and poured into saturated NaHCO_3_ solution (20–100 ml). The organic layer was separated and the aqueous phase extracted with CH_2_Cl_2_ (3 × 50 ml). The combined organic extracts were dried (MgSO_4_) and evaporated under reduced pressure. The product was used in the next step without further purification.

The corresponding diol was dissolved in dichloromethane (100 cm^3^) and perchloric acid was added dropwise (typically 3–6 drops). The resulting mixture was stirred overnight at room temperature. The residue was washed with saturated NaHCO_3_ (100 cm^3^) and re-extracted with dichloromethane or ethyl acetate (3 × 100 cm^3^). The combined extracts were dried over magnesium sulfate and the solvent removed under reduced pressure. All compounds were purified by column chromatography (silica).

#### 4,6-Diphenyl-4,6-dihydro-[1,3]dithiolo[4,5-*c*]furan-2-thione (**5**)

From the corresponding diol (0.93 g, 2.7 mmol) compound **5**, a brown solid, was isolated as a mixture of diastereomers (0.77 g, 87%). mp 153–155 °C; found: C, 61.76; H, 3.86; S, 28.68%. C_17_H_12_O_1_S_3_ requires C, 62.16; H, 3.68; S, 29.28%; *m/z* (CI) 329 (M^+^); δ_H_ (CDCl_3_) 7.40–7.20 (10H, m) and 6.10 (2H, s); 7.40–7.20 (10H, m) and 6.21 (2H, s); δ_C_ (CDCl_3_) 217.6, 141.3, 140.8, 137.8, 128.9, 126.4 and 84.9; *ν*_max_ (KBr)/cm^−1^ 1453, 1273, 1124, 1056, 1009, 749, 699 and 478.

#### 5-[Bis(4-methoxyphenyl)methyl]-2-thioxo-[1,3]dithiole-4-carbaldehyde (**6**)

From the corresponding diol (1.45 g, 3.6 mmol) compound **6** was obtained as a yellow flocculent solid (0.50 g, 36%). mp 60–62 °C; found: C, 58.66; H, 3.87; S, 25.20%. C_19_H_16_O_3_S_3_ requires C, 58.74; H, 4.15; S, 24.76%; *m/z* (CI) 389 (M^+^); δ_H_ (CDCl_3_) 9.61 (1H, s), 7.14 (4H, dd, *J* 6.7 and 1.9), 6.90 (4H, dd, *J* 6.7 and 2.2), 6.05 (1H, s) and 3.81 (6H, s); δ_C_ (CDCl_3_) 209.5, 178.1, 166.2, 159.3, 139.4, 132.3, 129.5, 114.5, 55.3 and 49.6; *ν*_max_ (KBr)/cm^−1^ 1664, 1608, 1511, 1249, 1178, 1077, 1031, 829 and 582.

#### 5-[Bis(2,4-dimethoxyphenyl)methyl]-4',5'-bis-methylsulfanyl-[2,2']bi{[1,3]dithiolylidene}-4-carbaldehyde (**20**)

From the corresponding diol (0.75 g, 1.2 mmol) compound **20** was obtained as a red oil (68 mg, 9%), *m/z* (EI) 610.0100 (M^+^, C_26_H_26_O_5_S_6_ requires 610.0104); δ_H_ (CDCl_3_) 9.60 (1H, s), 7.02 (2H, d, *J* 8.4), 6.43 (4H, m), 6.36 (1H, s), 3.86 (6H, s), 3.83 (6H, s), 2.46 (3H, s) and 2.43 (3H, s); δ_C_ (CDCl_3_) 180.0, 164.9, 160.8, 157.9, 132.3, 129.7, 128.4, 127.2, 121.7, 109.4, 104.4, 98.9, 56.0, 55.7, 37.1 and 19.5; *ν*_max_ (KBr)/cm^−1^ 1650, 1610, 1503, 1294, 1208 and 1033.

#### *N,N’*-Bis{5-[bis(2,4-dimethoxyphenyl)methyl]-2-thioxo-[1,3]dithiole-4-ylmethylene}ethan-1,2-diamine (**22**)

Compound **21** (0.50 g, 1.12 mmol) was dissolved in methanol (50 cm^3^). Ethylenediamine (0.06 g, 1.12 mmol) was added along with 1–3 drops of acetic acid with stirring. The solution was heated at reflux overnight. The resulting brown precipitate was recovered and washed successively with ice-cold methanol (50 cm^3^) and petroleum ether (50 cm^3^). Recrystallisation from dichloromethane/hexane gave the product as a pale brown crystalline solid (0.32 g, 62%). mp 213–215 °C; found: C, 57.19; H, 4.73; N, 3.12 %. C_44_H_44_N_2_O_8_S_6_ requires C, 57.43; H, 4.82; N, 3.04%; *m/z* (ES) 921 (M^+^); δ_H_ (CDCl_3_) 7.95 (2H, s), 6.95 (4H, d. *J* 8.5), 6.51 (4H, d, *J* 2.3), 6.46 (4H, dd, *J* 8.4 and 2.5), 3.82 (12H, s), 3.80 (12H, s) and 3.69 (4H, s); *ν*_max_ (KBr)/cm^−1^ 2935, 1612 and 1059.

#### 2,2’-Bis[(8-(2-thienyl)thieno[2,3-*f*][1,3]benzodithiolylidene)] (**25**)

8-(2-Thienyl)thieno[2,3-*f*][1,3]benzodithiole-2-thione **3** (0.377 g, 1.17 mmol) was suspended in the minimal amount of freshly distilled triethyl phosphite (<5 ml) under a nitrogen atmosphere. The suspension was placed into an oil bath pre-heated to 110 °C and heated under reflux with magnetic stirring for 20 h. A yellow-brown precipitate was collected on a Hirsch funnel and washed with copious amounts of hexane, then dissolved in chloroform and passed through a layer of silica gel eluting with chloroform until thin-layer chromatography indicated that the filtrate contained none of the product. Removal of solvent under reduced pressure afforded a bright yellow sparingly soluble solid, shown by ^1^H NMR to be a mixture of two isomers in the ratio 1:2.4, 80 mg, 12%, mp > 300 °C (decomp.); MALDI-TOF MS: 580; Accurate Mass calculated for C_26_H_12_S_8_ 579.8699 found 579.8707; ^1^H NMR (major isomer, 400 MHz, CDCl_3_) δ_H_ (ppm): 7.71 (1H, d, *J* = 0.7 Hz), 7.49 (1H, dd, *J* = 5.1 and 1.1 Hz), 7.34 (1H, d, *J* = 5.5 Hz), 7.26 (1H, dd, *J* = 3.5 and 1.1 Hz), 7.23 (1H, dd, *J* = 5.5 and 0.7 Hz), 7.19 (1H, dd, *J* = 5.1 and 3.5 Hz); (minor isomer, 400 MHz, CDCl_3_) δ_H_ (ppm): 6.68 (1H, d, *J* = 0.7 Hz), 7.52 (1H, dd, *J* = 5.1 and 1.2 Hz), 7.34 (1H, d, *J* = 5.5 Hz), 7.29 (1H, dd, *J* = 3.5 and 1.2 Hz), remaining minor isomer signals obscured in region 7.3–7.2; IR ν (cm^−1^) 3088, 1402, 1372, 1310, 1198, 1135, 1092, 842, 827, 715, 691; UV–vis, λ_max_ = 382 nm, ε = 2000; Anal. Calculated for C_26_H_12_S_8_: C, 53.76; H, 2.08; S, 44.16; found C, 53.56; H, 1.82; S, 44.14.

#### 8-(2-Thienyl)thieno[2,3-*f*][1,3]benzodithiole-2-one (**26**)

8-(2-Thienyl)thieno[2,3-*f*][1,3]benzodithiole-2-thione, **3** (0.171 g, 5.302 × 10^−4^ mol) was dissolved in a mixture of 3:1 by volume CH_2_Cl_2_:glacial acetic acid (100 ml) with magnetic stirring. Mercury (II) acetate (0.236 g, 7.423 × 10^−4^ mol) was added which caused an immediate lightening of the yellow solution and the formation of a white precipitate. The reaction was stirred at room temperature for 4 h then filtered through a layer of silica gel, washing with dichloromethane until the filtrate was colourless. The filtrate was concentrated to approximately 100 ml under reduced pressure then washed sequentially with water (75 ml), sodium hydrogen carbonate (saturated aqueous solution, 2 × 50 ml), water (100 ml), dried over magnesium sulfate and filtered. The solvent was removed under reduced pressure to yield a straw-coloured solid which was purified by recrystallisation from dichloromethane and petroleum ether 40/60, to give **26** as straw needles, 0.145 g, 89%, mp 118–124 °C; EI+ MS (M+) 306; Accurate mass calculated for C_13_H_6_S_4_O 305.9296, found 305.9292; ^1^H NMR (300 MHz, CDCl_3_) δ_H_ (ppm): 7.95 (1H, d, *J* = 0.7 Hz), 7.53 (1H, dd, *J* = 5.1 and 1.2 Hz), 7.45 (1H, d, *J* = 5.6 Hz), 7.34 (1H, dd, *J* = 5.6 and 0.7 Hz), 7.27 (1H, dd, *J* = 3.6 and 1.2 Hz), 7.20 (1H, dd, *J* = 5.1 and 3.6 Hz); ^13^C NMR (75 MHz, CDCl_3_) δ_C_ (ppm): 189.6, 138.8, 138.8, 138.6, 131.0, 129.1, 128.5, 127.7, 127.6, 127.3, 125.5, 123.2, 116.5; IR ν (cm^−1^) 3102, 1651, 1403, 1370, 1068, 875, 852, 828, 697; Anal. calculated for C_13_H_6_S_4_O: C, 50.95; H, 1.97; S, 41.85; found: C, 50.52, H, 1.62; S, 42.66.

#### 5,6-ethylenedithio-4-(thiophen-2-yl)benzo[*b*]thiophene (**27**)

8-(2-Thienyl)thieno[2,3-*f*][1,3]benzodithiole-2-one **26** (0.200 g, 6.52 × 10^−4^ mol) was dissolved in anhydrous tetrahydrofuran (15 ml) under a nitrogen atmosphere with magnetic stirring. Separately, a methanolic solution of sodium methoxide (0.718 mol·L^−1^) was prepared by adding sodium (0.428 g, 0.0186 mol) to anhydrous methanol (15 ml) under a nitrogen atmosphere. 2.0 ml of this sodium methoxide solution (2.2 equivalents (0.001434 mol) was added by syringe to the THF solution. The mixture was stirred at room temperature for 1.5 h, then 1,2-dibromoethane (0.058 ml, 0.126 g, 6.700 × 10^−4^ mol) was added and the reaction stirred at room temperature for 16 h, during which time a white precipitate formed. The mixture was added to water (50 ml) and extracted with dichloromethane (3 × 100 ml). The combined organic extracts were dried over magnesium sulfate, filtered, and the solvent was removed under reduced pressure. The resulting yellow solid was purified by column chromatography on silica gel eluting with 1:1 (v/v) dichloromethane:petroleum ether 40/60 to yield a bright yellow waxy solid 0.112 g, 56%, mp 57–61 °C; APCI +ve MS (M + 1) 307; Accurate mass calculated for C_14_H_10_S_4_ 305.9660, found 305.9666; ^1^H NMR (400 MHz, CDCl_3_) d_H_ (ppm): 7.83 (1H, d, *J* = 0.7 Hz), 7.51 (1H, dd, *J* = 5.1 and 1.2 Hz), 7.29 (1H, d, *J* = 5.6 Hz), 7.20 (1H, dd, *J* = 5.1 and 3.5 Hz), 7.11 (1H, dd, *J* = 3.5 and 1.2 Hz), 7.02 (1H, dd, *J* = 5.6 and 0.7 Hz), 3.33 (2H, m), 3.16 (2H, m); ^13^C NMR (100 MHz, CDCl_3_) δ_C_ (ppm): 139.4, 138.5, 136.9, 131.1, 129.2, 129.0, 127.2, 126.8, 126.4, 123.6, 122.7, 114.9, 30.9, 30.7; IR ν (cm^−1^) 3100, 2916, 1559, 1406, 1365, 1312, 1288, 1194, 1138, 853, 824. 769, 702; UV–vis, λ_max_ = 376 nm, ε = 8000.

#### Tetrabutylammonium bis[(4-thiophen-2-yl)benzo[*b*]thiophene-5,6-bis(thiolato)]nickel(II) (**28**)

To a solution of 8-(2-thienyl)thieno[2,3-*f*][1,3]benzodithiole-2-thione **3** (0.092 g, 2.85 × 10^−4^ mol) in dry THF (10 ml) under a nitrogen atmosphere, was added sodium ethoxide (0.1 M aqueous solution, 2.85 ml, 2.85 × 10^−4^ mol) and the mixture heated under reflux for 1 h. Tetrabutylammonium bromide (0.097 g, 3.01 × 10^−4^ mol) and nickel (II) chloride hexahydrate (0.036 g, 1.51 × 10^−4^ mol) were then added and the mixture was stirred magnetically for 18 h then filtered. The filtrate was concentrated to a few ml under reduced pressure, and addition of petroleum ether 40/60 afforded a dark green precipitate which was recrystallised from dichloromethane/hexane, 0.103 g, 40%, mp 215–216 °C; ^1^H NMR (major isomer, 400 MHz, CDCl_3_) δ_H_ (ppm): 8.22 (relative integral 2.5, broad multiplet), 8.07 (relative integral 7.8, broad d), 7.51 (relative integral 1.4, broad multiplet), 4.69 (relative integral 11.0, s), 4.51 (relative integral 1.1, broad s), 3.88 (relative integral 1.0, broad s), 3.13 (relative integral 2.1, v. broad s), 1.63 (relative integral 6.1, broad s), 1.32 (relative integral 3.0, broad s), 1.26 (relative integral 1.4, s), 0.90 (relative integral 5.4, broad m); IR ν (cm^−1^) 3417, 3091, 2956. 1618, 1466, 1311, 1286, 851, 822, 699; UV–vis, λ_max_ = 901 nm, ε = 12000.
